# Non-Invasive Brain Stimulation in Older Inpatients with Depression: A Real-World Comparison of Repetitive Transcranial Magnetic Stimulation (rTMS) and Transcranial Direct Current Stimulation (tDCS) on Depressive Symptoms and Functional Recovery

**DOI:** 10.3390/biomedicines14030650

**Published:** 2026-03-13

**Authors:** Michele Prato, Barbara Barbini, Filippo Frizzi, Matteo Carminati, Greta Verri, Sebastiano Busseni Cantoni, Thomas Kafka, Raffaella Zanardi, Cristina Colombo

**Affiliations:** 1 Department of Clinical Neurosciences, Vita-Salute San Raffaele University, 20127 Milan, Italy; prato.michele@hsr.it (M.P.); frizzi.filippo@hsr.it (F.F.); verri.greta@hsr.it (G.V.); busseni.sebastiano@hsr.it (S.B.C.); t.kafka@studenti.unisr.it (T.K.); zanardi.raffaella@hsr.it (R.Z.); colombo.cristina@hsr.it (C.C.); 2 Department of Psychiatry, Mood Disorder Unit, Scientific Institute IRCCS Ospedale San Raffaele, 20127 Milan, Italy; carminati.matteo@hsr.it

**Keywords:** late-life depression (LLD), repetitive transcranial magnetic stimulation (rTMS), transcranial direct current stimulation (tDCS), non-invasive brain stimulation (NIBS), global functioning

## Abstract

**Background:** Non-invasive brain stimulation (NIBS) is increasingly used as an adjunctive option in late-life depression (≥60 years), a condition frequently complicated by multimorbidity and incomplete response to standard treatments. Comparative real-world evidence between repetitive Transcranial Magnetic Stimulation (rTMS) and transcranial Direct Current Stimulation (tDCS), particularly including functional outcomes, remains limited. **Methods:** We conducted a retrospective, naturalistic comparative study of 104 depressed inpatients (≥60 years), either unipolar or bipolar, treated with rTMS (n = 48) or tDCS (n = 56) as part of routine care. Depression severity was assessed with the 21-item Hamilton Depression Rating Scale (HDRS21) at baseline, 2 weeks, and 1 month; response was defined as ≥50% HDRS21 score reduction and remission as HDRS21 < 7 at 1 month. Global Assessment of Functioning (GAF) was assessed at admission and discharge (baseline and 1 month). Longitudinal changes were examined using covariate-adjusted mixed-effects models; categorical outcomes were compared using χ^2^ tests. Propensity score matching was applied as an additional approach to reduce confounding due to the observational design. **Results:** At 1 month, response and remission rates were significantly higher in the rTMS group than in the tDCS group (87.5% vs. 55.4%, *p* < 0.001; 62.5% vs. 41.1%, *p* = 0.047, respectively). rTMS showed greater HDRS21 score reductions at 2 weeks and 1 month (Time × Treatment, *p* < 0.001). GAF scores significantly improved over time in both groups (Time effect, *p* < 0.001) without between-technique differences (Time × Treatment, *p* = 0.56), and GAF scores did not differ by response/remission status. **Conclusions:** In this cohort of inpatients aged ≥ 60 years with depressive episodes, rTMS was associated with greater short-term reductions in HDRS21 scores compared with tDCS, whereas both modalities showed comparable improvements in GAF from admission to discharge.

## 1. Introduction

Late-life depression (LLD), conventionally defined as Major Depressive Disorder (MDD) in adults aged ≥ 60 years [1], is a growing clinical and public-health challenge. Depressive disorders in older adults are common across both community and clinical settings, with prevalence increasing in more medically and functionally burdened populations; clinically significant depressive symptoms are even more frequent, and also women remain disproportionately affected in later life [2,3,4,5,6,7]. At the same time, multimorbidity, cognitive change, and polypharmacy complicate both detection and management, while heterogeneity in underlying factors such as cerebrovascular burden, executive dysfunction, and medical comorbidities further limits the applicability of standardized treatment strategies in this age group [1,8]. Importantly, LLD is also a major driver of disability and loss of autonomy, and this burden is closely intertwined with cognitive decline in aging. Longitudinal and meta-analytic evidence indicates that depression in later life is associated with accelerated cognitive decline and increased risk of incident dementia, supporting the need to evaluate outcomes beyond symptom severity alone [9,10,11,12,13].

In this context, non-invasive brain stimulation (NIBS) is increasingly used as an augmentation strategy when response to standard antidepressant treatments is insufficient. Repetitive Transcranial Magnetic Stimulation (rTMS), which uses magnetic pulses delivered through a scalp coil to modulate cortical activity, has a robust evidence of efficacy in adult depression and a growing literature in older adults, with meta-analyses supporting superiority over sham and acceptable tolerability [14,15]. Transcranial Direct Current Stimulation (tDCS), which applies low-intensity direct current through scalp electrodes to modulate cortical excitability, has shown antidepressant efficacy versus sham in individual patient meta-analysis of adult trials [16] and has emerging evidence specifically in older cohorts, including an individual participant-data meta-analysis in LLD and a randomized trial in vascular depression [17,18]. Both techniques commonly target the left dorsolateral prefrontal cortex (DLPFC), a core region within fronto-limbic and cognitive control networks implicated in mood regulation. Converging neuroimaging evidence in depression supports the relevance of the DLPFC as a therapeutic target, given its role in executive control, emotion regulation, and the top-down modulation of limbic regions [19,20,21]. In this framework, neuromodulation of the left DLPFC is thought to promote antidepressant effects by modifying cortical excitability and network-level connectivity in circuits involved in depressive symptom generation and maintenance [22,23,24,25]. Direct comparative evidence between rTMS and tDCS in late-life depression remains scarce. To our knowledge, no naturalistic real-world study has directly compared these techniques in older patients with depressive disorders, and available comparative inferences rely largely on pooled or network-based indirect analyses of separate trials [26,27,28,29]. This leaves uncertainty about comparative effectiveness in clinically complex inpatient cohorts that are often underrepresented in explanatory trials.

While clinician-rated depression severity remains the primary target in antidepressant and NIBS studies, symptomatic improvement is not synonymous with functional recovery. Functional gains may lag symptom change, and residual disability can persist even when depressive symptoms improve [13,30]. For pragmatic assessment in routine care, the Global Assessment of Functioning (GAF) has been widely recorded over decades and is commonly available in inpatient records, enabling admission-to-discharge comparisons in real-world datasets [31,32,33]. Although it is a global measure, work on the GAF supports the notion that symptom severity and functioning may not map perfectly, which makes it useful as a complementary outcome alongside symptom scales [34].

The present study aimed to provide a retrospective, real-world, head-to-head comparison of rTMS and tDCS in older psychiatric inpatients (≥60 years) experiencing a Major Depressive Episode (MDE) in the context of MDD or Bipolar Disorder (BD), by assessing both depressive symptom trajectories (21-item Hamilton Depression Rating Scale, HDRS21) and global functioning (GAF) during hospitalization. The primary endpoint was change in HDRS21, and the secondary endpoint was change in GAF, to describe functional change alongside symptomatic trajectories. By providing head-to-head evidence from routine inpatient care, this study aims to inform technique selection and clinical expectations for outcomes that matter to patients and clinicians.

## 2. Materials and Methods

### 2.1. Study Design and Setting

This retrospective, naturalistic comparative study was conducted at the Mood Disorder Unit of IRCCS San Raffaele Hospital (Milan, Italy). Clinical records from January 2019 to December 2024 were retrospectively reviewed. We analyzed routinely collected clinical records of 104 older inpatients (≥60 years) with depressive episodes who received NIBS as part of clinical care. All participants provided written informed consent, and the study protocol received approval from the local Ethics Committee of IRCCS San Raffaele Hospital, Milan (protocol code 10-06-SO; date of approval: 13 December 2018).

### 2.2. Participants

Eligible participants were older inpatients (age ≥ 60 years at baseline) with a DSM-5 (Diagnostic and Statistical Manual of Mental Disorders, 5th edition) diagnosis of MDD or BD [35] and a current MDE at admission. Treatment allocation (rTMS vs. tDCS) was based on clinician discretion. All patients were non-responders to at least one adequate antidepressant trial during the current episode [36,37]. Exclusion criteria reflected standard safety practice and included: contraindications to rTMS/tDCS (e.g., implanted metallic/electrical devices or intracranial metallic implants); current or past epilepsy (or relevant history when clinically considered); major neurological disease or prior neurosurgical interventions; unstable or severe medical illness; active psychotic symptoms; recent (≤6 months) alcohol/substance abuse; and pregnancy. No patient received both techniques.

### 2.3. Interventions

rTMS was delivered once daily, 5 days/week for 4 weeks (total 20 sessions, 60,000 pulses). Each session consisted of 75 trains of 10 Hz stimulation targeting the left DLPFC at 120% of the resting motor threshold; train duration was 4 s, with an inter-train interval ranging from 11 to 26 s according to tolerability. Stimulation was delivered using a Magstim Rapid2 (The Magstim Company Limited, Wales, UK) with a 70 mm air-cooled double coil, and treatment was administered and monitored by trained clinical staff in accordance with international safety recommendations [38,39]. tDCS was delivered 5 days/week for 4 weeks (total 20 sessions) using an E.M.S. BrainSTIM© stimulator (EMS Biomedical GmbH, Korneuburg, Austria) with two 5 × 5 cm saline-soaked sponge electrodes (0.9% NaCl). A constant current of 2 mA was applied for 30 min per session, with the anode positioned over F3 (10–20 EEG system; left DLPFC) and the cathode over the contralateral supraorbital area. Concomitant psychopharmacological treatments were continued as clinically indicated throughout hospitalization; patients were required to be on a stable psychopharmacological regimen for at least 4 weeks before NIBS initiation, and treatments were not modified during the rTMS/tDCS treatment course. Medications known to substantially affect cortical excitability or seizure threshold (e.g., antiepileptics) were avoided when clinically feasible, consistent with standard safety practice [38,39]. No serious adverse events were documented in the available clinical records; safety monitoring followed routine practice and international recommendations [38,39].

All patients participated in the unit’s standard inpatient psychiatric rehabilitation program, delivered by psychiatric rehabilitation technicians and consisting of daily structured activities (group and individual sessions) including skills training, occupational activities, and psychoeducation throughout the admission (approximately 1 month).

### 2.4. Outcomes

Depression severity was assessed using the HDRS21 at baseline, 2 weeks and at 1 month [40]. Response was defined as a reduction of ≥50% in HDRS21 total score from baseline, and remission as HDRS21 < 7, both evaluated at 1 month, in line with commonly used operational criteria for depression outcomes [41,42]. Global functioning was assessed using the GAF scale at admission (baseline) and discharge (approximately 1 month), as described in DSM-IV-TR Axis V (Diagnostic and Statistical Manual of Mental Disorders, Fourth Edition, Text Revision; Axis V: Global Assessment of Functioning) [43] and supported by structured rating guidance [32]. HDRS21 and GAF were rated by trained psychiatrists as part of routine clinical assessments.

### 2.5. Statistical Analysis

Baseline sociodemographic and clinical characteristics were summarized using descriptive statistics; between-group differences were tested using *t*-tests for continuous variables and χ^2^ tests (or Fisher’s exact tests when appropriate) for categorical variables.

For the primary categorical outcomes, response and remission at 1 month were compared between rTMS and tDCS using χ^2^ tests, and effect sizes were reported as relative risks (RRs) with 95% confidence intervals (CIs). To explore whether differences in response and remission rates were influenced by potential confounders, multivariable logistic regression models were fitted, including treatment protocol, age, episode duration, diagnosis (MDD vs. BD), and antidepressant class (Selective Serotonin Reuptake Inhibitors, SSRIs; Serotonin-Norepinephrine Reuptake Inhibitors, SNRIs; Tricyclic Antidepressants, TCAs), as well as concomitant psychotropic treatments (mood stabilizers, antipsychotics, and benzodiazepines); effect sizes were reported as odds ratios (ORs) with 95% confidence intervals (CIs).

Longitudinal changes in HDRS21 scores (baseline, 2 weeks, 1 month) were examined using linear mixed-effects models with random intercepts for participants. Fixed effects included Time, Treatment, and the Time × Treatment interaction. Adjusted models additionally included age, episode duration, the episode duration × Treatment interaction, age at onset, diagnosis (MDD vs. BD), the diagnosis × Treatment interaction, antidepressant class (SSRI vs. SNRI vs. TCA), and concomitant psychotropic treatments (mood stabilizers, antipsychotics, and benzodiazepines). Normality of model residuals and random effects was assessed using the Shapiro–Wilk test and by visual inspection of Q–Q plots. Homoscedasticity was evaluated using a nonparametric dispersion test and by inspection of residuals-versus-fitted plots. The linearity of continuous covariates was assessed graphically.

Given the observational nature of the study, a 1:1 propensity score matching without replacement was applied to reduce confounding. Propensity scores were estimated using a logistic regression model including age, baseline HDRS21 severity, episode duration, diagnosis, and antidepressant therapy. Nearest-neighbor matching was then used to create balanced treatment groups, and the same adjusted linear mixed-effects model was re-fitted in the matched sample. A caliper width of 0.2 standard deviations of the logit of the propensity score was utilized; covariate balance was assessed through standardized mean differences (SMDs).

Functional change in GAF was analyzed using linear mixed-effects models with random intercepts for participants. Fixed effects included Time, Treatment, and the Time × Treatment interaction. Adjusted models additionally included age, episode duration, age at onset, diagnosis (MDD vs. BD), the diagnosis × Treatment interaction, antidepressant class (SSRI vs. SNRI vs. TCA), and concomitant psychotropic treatments (mood stabilizers, antipsychotics, and benzodiazepines). The same model was also re-fitted in the matched sample. All tests were two-tailed with α = 0.05. Analyses were conducted in R (version 4.5.1) [44].

## 3. Results

A total of 104 patients were included, comprising 19 males and 85 females, with a mean age of 69.12 years. Of these, 48 received rTMS and 56 received tDCS, according to clinician discretion. Sociodemographic and clinical characteristics are reported in Table 1. The sample included 85 patients with MDD and 19 with BD. Across the sample, antidepressant treatment included SSRIs (n = 57), SNRIs (n = 23), and TCAs (n = 24); concomitant psychotropic treatments included mood stabilizers (n = 20), antipsychotics (n = 18), and benzodiazepines (n = 82). No significant between-group differences were observed in baseline sociodemographic or clinical variables, except for age, with patients treated with tDCS being significantly older than those treated with rTMS.

### 3.1. Primary Outcomes: Response and Remission

**Figure 1:**
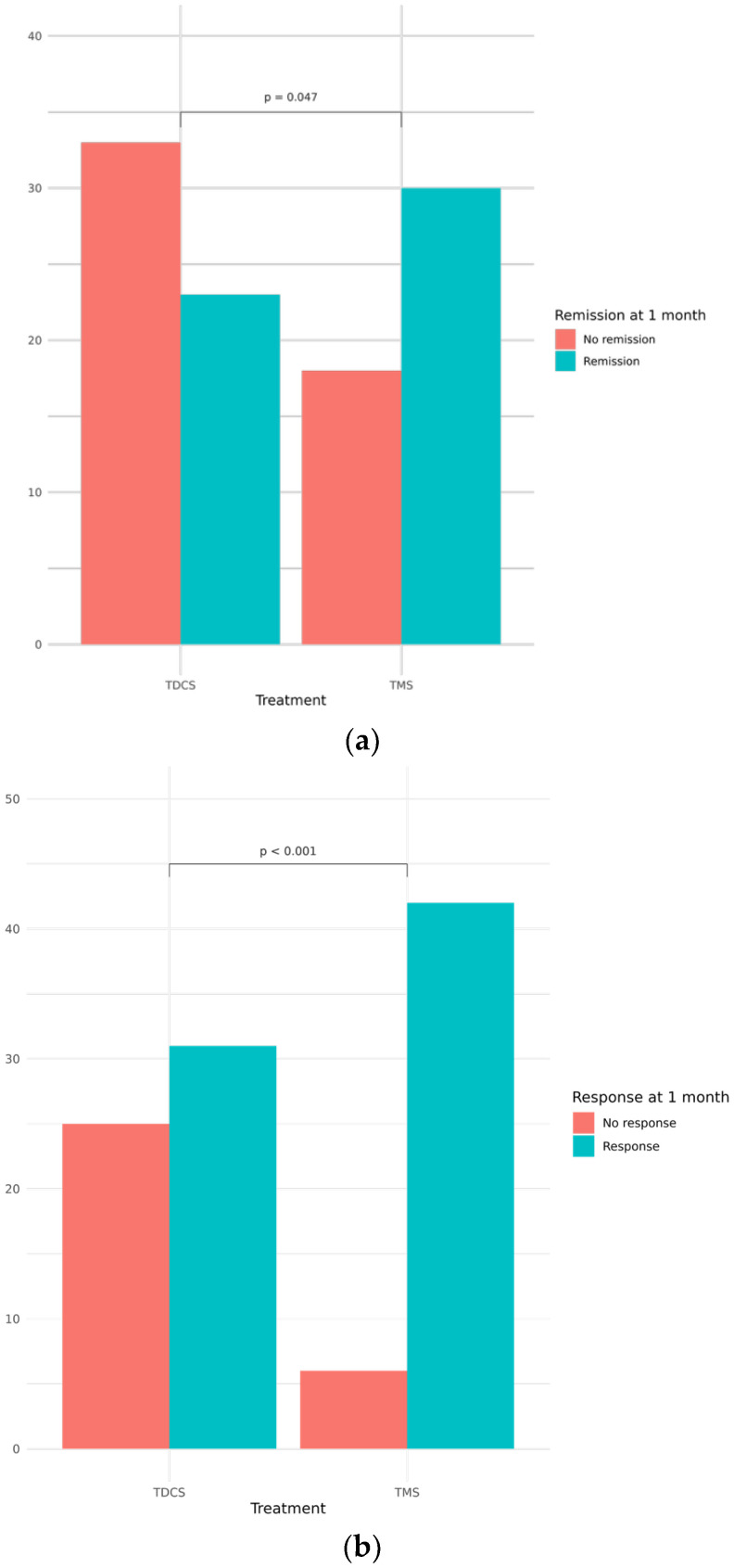
(**a**) Absolute count of patients achieving vs. non achieving remission at 1 month follow-up, stratified by treatment protocol. Remission is defined as HDRS21 < 7; (**b**) absolute frequency of responders vs. non-responders at 1 month follow-up, stratified by treatment protocol. Response is defined as a ≥50% reduction in HDRS21 score.

As shown in Figure 1a,b, response (defined as a ≥50% reduction in HDRS21 score) and remission (HDRS21 < 7) were assessed 1 month after treatment initiation. At 1 month, the response rate was significantly higher in the rTMS group than in the tDCS group (87.5% vs. 55.4%, χ^2^ = 11.27, *p* < 0.001, RR = 1.58, 95% CI 1.22–2.05). Remission rates also favored rTMS (62.5% vs. 41.1%, χ^2^ = 3.97, *p* = 0.047, RR = 1.52, 95% CI 1.04–2.23). No significant association emerged between the examined baseline variables and either response or remission.

To explore whether between-group differences in response and remission were influenced by potential confounders, multivariable logistic regression models were fitted, including age, episode duration, diagnosis (MDD vs. BD), antidepressant class (SSRIs vs. SNRIs vs. TCAs), and concomitant psychotropic treatments (mood stabilizers, antipsychotics, and benzodiazepines). rTMS was strongly associated with higher odds of response (OR = 7.84, 95% CI 2.34–31.77, *p* = 0.0005), whereas longer episode duration showed a small but significant negative association with response (OR = 0.89, 95% CI 0.77–0.94, *p* = 0.03). Regarding remission, the estimated odds ratio for treatment was in the expected direction (OR = 2.23, 95% CI 0.88–6.19), although the association did not reach statistical significance (*p* = 0.08). None of the predictors in the model was statistically significant, and the likelihood ratio test did not indicate a significant improvement of the full model over the null model, suggesting limited statistical power for this analysis.

### 3.2. Continuous Outcomes: Longitudinal HDRS21 Trajectories

To explore differences in longitudinal symptom change between rTMS and tDCS, a linear mixed-effects model was fitted, including age, episode duration, age at onset, diagnosis (MDD vs. BD), antidepressant class (SSRIs vs. SNRIs vs. TCAs), and concomitant psychotropic treatments (mood stabilizers, antipsychotics, and benzodiazepines), with a random intercept for subject to account for within-subject correlation. The normality of model residuals and random effects was supported by the Shapiro–Wilk test (*p* = 0.53 and *p* = 0.45, respectively) and by visual inspection of Q–Q plots. Homoscedasticity was supported by a nonparametric dispersion test (*p* = 0.65) and inspection of residuals-versus-fitted plots, and linearity of continuous covariates was assessed graphically.

**Figure 2:**
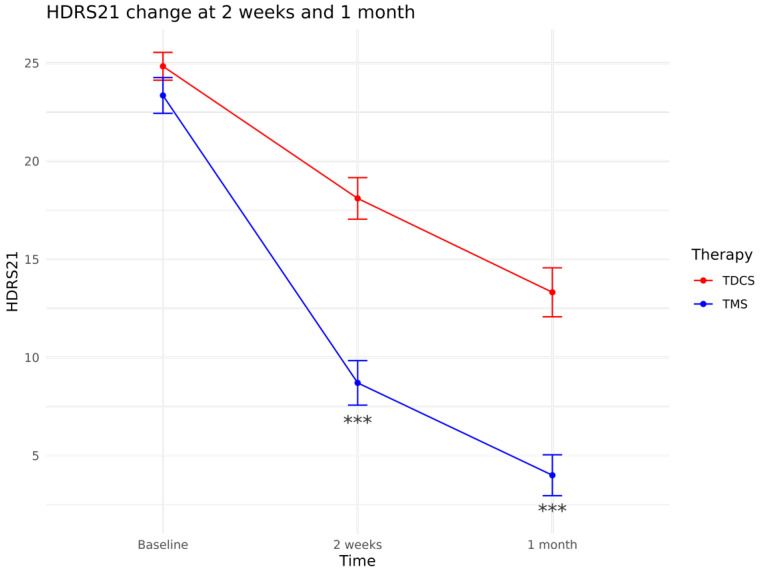
Means and 95% confidence intervals HDRS21 scores in the rTMS and tDCS protocol groups at baseline, at 2 weeks and at 1 month follow-up. *** *p* value < 0.05.

Patients treated with rTMS showed a greater reduction in HDRS21 scores both at 2 weeks (Time × Treatment: β = −6.12, *p* < 0.001) and at 1 month (Time × Treatment: β = −4.46, *p* < 0.001). Longer episode duration was associated with a small borderline significant reduction in treatment effectiveness (β = 0.24, *p* = 0.05), irrespective of treatment protocol (episode duration × Treatment: β = −0.23, *p* = 0.31); no difference was observed between patients with MDD vs. BD (Treatment × diagnosis: β = 0.92, *p* = 0.73). Figure 2 illustrates HDRS21 trajectories by treatment group.

Given the observational nature of the study and the baseline age difference between treatment groups, a 1:1 propensity score matching without replacement was applied to improve comparability between groups. Propensity scores were estimated using a logistic regression model including age, baseline HDRS21 score, episode duration, diagnosis, and concomitant psychotropic treatments (antidepressant class, mood stabilizer therapy, antipsychotics, and benzodiazepines). Nearest-neighbor matching yielded a matched sample of 21 patients treated with rTMS and 21 treated with tDCS; baseline characteristics of the matched sample are reported in Table 2. A caliper width of 0.2 standard deviations of the logit of the propensity score was used, and covariate balance between groups was assessed using SMDs, which are reported in Table 1 and Table 2.

A substantial improvement in covariate balance was achieved, although mild imbalance persisted in some of the covariates.

The same adjusted linear mixed-effects model was then re-fitted in the matched sample. Findings were consistent with those observed in the full sample, with rTMS showing a greater reduction in HDRS21 scores both at 2 weeks (Time × Treatment: β = −7.52, *p* < 0.001) and at 1 month (Time × Treatment: β = −7.19, *p* < 0.001). Longer episode duration was again associated with slightly lower treatment effectiveness (β = 0.38, *p* = 0.02), irrespective of treatment protocol (episode duration × Treatment: β = −0.47, *p* = 0.11); the interaction between diagnosis (MDD vs. BD) and treatment was not statistically significant (β = −0.64, *p* = 0.87).

### 3.3. Functional Outcomes: GAF

**Figure 3:**
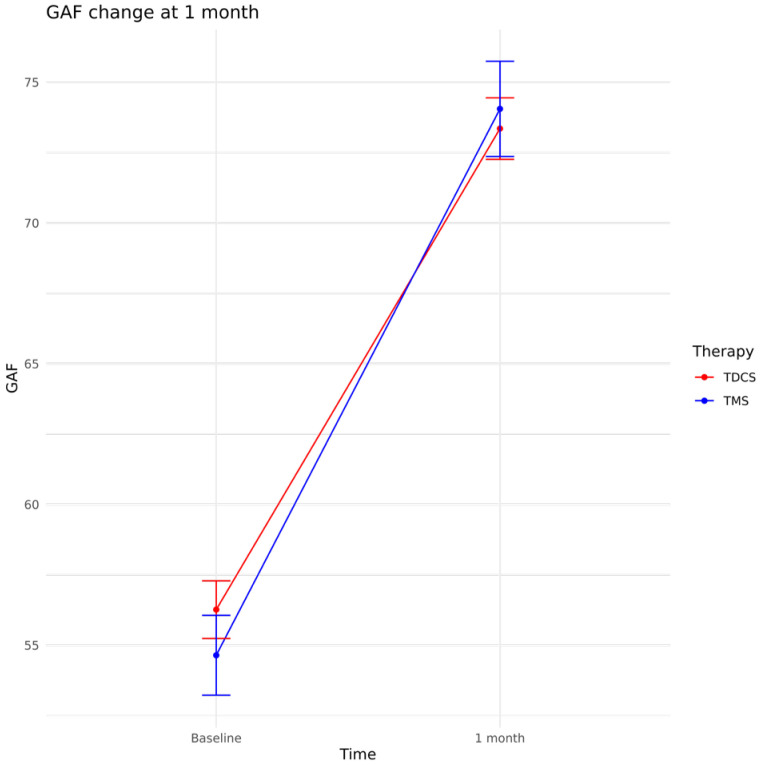
Means and 95% confidence interval GAF scores in the TMS and tDCS protocol groups at baseline, at 2 weeks and at 1 month follow-up.

Interestingly, despite the marked between-group difference in depressive symptom reduction, both treatment protocols were associated with significant improvement in global functioning at 1 month, as measured by the GAF (Time effect: β = 17.07, *p* < 0.001), with no between-group difference (Time × Treatment: β = −0.83, *p* = 0.65), after adjustment for age, episode duration, age at onset, diagnosis (MDD vs. BD), antidepressant class (SSRIs vs. SNRIs vs. TCAs), and concomitant psychotropic treatments (mood stabilizers, antipsychotics, and benzodiazepines). Re-fitting the same model in the matched sample yielded comparable results (Time effect: β = 17.24, *p* < 0.001; Time × Treatment: β = −0.61, *p* = 0.81), and none of the covariates reached statistical significance. Patient trajectories by treatment protocol are shown in Figure 3.

In addition, patients achieving remission or response did not show significantly higher GAF scores than those who did not (remission: t = −0.94, *p* = 0.35; response: t = −0.16, *p* = 0.84).

## 4. Discussion

In this retrospective, naturalistic study of older psychiatric inpatients with depressive episodes, rTMS was associated with greater short-term symptomatic improvement than tDCS. Response and remission rates at 1 month were higher in the rTMS group, and HDRS21 scores decreased more rapidly over time; importantly, the latter finding remained consistent in the propensity score-matched analyses. By contrast, global functioning improved during hospitalization in both groups, with no significant between-technique differences in GAF change and no clear separation of discharge GAF scores by response/remission status. Because direct rTMS–tDCS comparisons remain scarce, and much of the comparative literature relies on indirect evidence, these data add pragmatic head-to-head information from routine inpatient care [26,27,28,29].

In our cohort, rTMS was associated with clinically meaningful symptom improvement in LLD, consistent with meta-analyses and randomized studies supporting antidepressant efficacy and acceptable tolerability of rTMS in older adults [14,15,45]. Conversely, the response and remission rates observed in our rTMS group were higher than those reported in the majority of controlled trials, and this warrants careful interpretation. Several factors may have contributed. First, treatment was delivered within an intensive inpatient setting that included structured rehabilitation, daily monitoring, and multidisciplinary care, all of which may enhance early clinical improvement. Second, non-specific contextual influences—including patient expectations and therapeutic context effects—may contribute to short-term symptom change in depression treatment more broadly, and could be particularly relevant in highly structured inpatient care [46,47]. Finally, although the adjusted analyses did not identify a broad effect of the examined clinical and sociodemographic covariates on treatment outcome, patient-level factors may still influence treatment selection and outcomes in naturalistic designs, particularly when not all relevant dimensions of clinical complexity are captured. This interpretation is consistent with real-world registry evidence showing that clinical and sociodemographic variables may contribute to variability in TMS response and remission rates [48].

Notably, longer episode duration was associated with lower treatment effectiveness in both rTMS and tDCS groups, suggesting that episode chronicity may negatively influence short-term symptomatic improvement in LLD, in line with previous literature identifying current episode duration as a clinically relevant predictor of treatment outcome in depression [49].

Findings for tDCS should be interpreted within the current evidence base, which remains more heterogeneous in older adults. In adult major depression, individual patient-data meta-analysis supports antidepressant efficacy versus sham, although average effects are generally modest and heterogeneous [16]. In LLD, the available evidence suggests a likely antidepressant effect, but the number of controlled studies remains limited [17], and specific subgroups such as vascular depression may have partially different clinical and biological profiles [18,50,51,52]. In our cohort, both techniques were delivered in the same inpatient pathway and both groups improved clinically, but rTMS was associated with larger short-term symptom reductions.

From a neurobiological perspective, this comparison is clinically meaningful because both rTMS and tDCS targeted the left DLPFC, a region involved in cognitive control, emotion regulation, and executive functioning, domains frequently affected in LLD [1,8]. LLD is often associated with fronto-subcortical and cerebrovascular changes that may alter network-level regulation of mood and cognition [50,51,52]. In this framework, both techniques aim to modulate prefrontal circuits, but they do so through different stimulation mechanisms and with different focality/intensity profiles, which may contribute to the different symptomatic trajectories observed in our sample. This interpretation is consistent with the broader depression literature showing that DLPFC-centered neuromodulation can influence dysfunctional cortico-limbic networks implicated in depressive symptoms [20,21].

Importantly, the GAF remains a widely used and clinically interpretable summary of overall functioning in inpatient records and has been shown to be sensitive to change, particularly for admission-to-discharge evaluation [31,32,53]. At the same time, as a global measure, it does not precisely separate symptom severity from functioning and may be less sensitive to differences between subgroups defined primarily by symptom response at discharge [33,34,54]. In this context, the absence of GAF separation by response/remission may reflect both the multidimensional nature of functional recovery during hospitalization and the short follow-up interval. These findings support assessing both depressive symptoms and functioning when evaluating outcomes in LLD. Future studies should include more specific functional measures (e.g., WHODAS 2.0 or domain-based disability scales) and longer follow-up to determine whether early symptomatic differences translate into more sustained functional advantages [13,33].

Several limitations should be acknowledged. Because treatment allocation was based on clinical judgment rather than randomization, residual confounding cannot be fully excluded, although the analyses included multivariable adjustment and propensity score matching. Moreover, routinely collected records did not include standardized measures of cognitive impairment, vascular burden, or other markers of illness complexity, limiting mechanistic interpretation in LLD. Finally, outcomes were assessed over a relatively short period of 1 month, and longer follow-up is needed to establish whether early symptomatic differences translate into sustained functional advantages.

## 5. Conclusions

In older inpatients with depressive episodes, both rTMS and tDCS were feasible in routine clinical care and were associated with improvement during hospitalization. A between-technique difference was observed for depressive symptoms, with greater short-term improvement in the rTMS group, whereas no between-technique differences emerged for global functioning (GAF). This real-world head-to-head comparison adds clinically relevant evidence to a field in which direct comparisons between rTMS and tDCS remain scarce. Prospective studies with longer follow-up, richer characterization of cognitive and vascular features, and more specific functional outcome measures are needed to clarify which patients with LLD benefit most from each neuromodulation approach.

## Figures and Tables

**Table 1:** Summary of sociodemographic and clinical variables of the sample.

	rTMS (n = 48)	tDCS (n = 56)	Total (n = 104)	*p*-Value	SMD
Sex (male)	12; 25.00%	7 12.50%	43; 41.35%	0.16 ^a^	0.434
Diagnosis (MDD)	38; 79.17%	47; 83.93%	85; 81.73%	0.71 ^a^	−0.002
Age (years; mean ± SD)	65.29 ± 5.21	72.39 ± 5.75	69.12 ± 6.53	<0.001 ^b^	−1.311
Age at onset (years; mean ± SD)	43.30 ± 14.66	47.48 ± 15.19	45.93 ± 15.05	0.21 ^b^	−0.205
Current episode duration (weeks; mean ± SD)	6.64 ± 4.22	5.31 ± 4.31	6,84 ± 4.59	0.05 ^b^	0.379
Antidepressants (SSRIs; SNRIs; TCAs)	28; 9; 11 58.33%; 18.75%; 22.92%	29; 14; 13 51.79%; 25.00%; 23.21%	57; 23; 24 54.81%; 22.12%; 23.08%	0.34 ^b^	0.308
Mood stabilizers (yes)	12; 25.00%	8; 16.67%	20; 19.23%	0.26 ^b^	0.102
Antipsychotics (yes)	12; 25.00%	6; 10.70%	18; 17.31%	0.10 ^b^	0.212
Benzodiazepines (yes)	37; 77.08%	45; 80.36%	82; 78.85%	0.69 ^b^	−0.020
HDRS21 at baseline	23.47 ± 4.23	24.84 ± 4.59	24.34 ± 4.49	0.22 ^b^	−0.495
HDRS21 at 2 weeks	9.00 ± 4.39	18.11 ± 6.87	14.79 ± 7.49	<0.001 ^b^	−1.449
HDRS21 at 1 month	4.84 ± 3.87	13.32 ± 8.10	10.24 ± 7.98	<0.001 ^b^	−1.069
GAF at baseline	55.13 ± 6.42	56.27 ± 6.64	55.74 ± 6.53	0.56 ^b^	−0.163
GAF at 1 month	72.58 ± 8.49	73.36 ± 7.09	73.00 ± 7.74	0.74 ^b^	−0.020

^a^ Mann–Whitney; ^b^ Chi-Squared; SMD: standardized mean difference; rTMS: repetitive Transcranial Magnetic Stimulation; tDCS: transcranial Direct Current Stimulation; MDD: Major Depressive Disorder; SD: standard deviation; HDRS21: 21-item Hamilton Depression Rating Scale; GAF: Global Assessment of Functioning; SSRIs: Selective Serotonin Reuptake Inhibitors; SNRIs: Serotonin-Norepinephrine Reuptake Inhibitors; TCAs: Tricyclic Antidepressants.

**Table 2:** Summary of sociodemographic and clinical variables of the sample after propensity-based matching.

	rTMS (n = 21)	tDCS (n = 21)	Total (n = 42)	*p*-Value	SMD
Sex (male)	4; 19.05%	3; 14.29%	7; 16.67%	1.00 ^b^	0.128
Diagnosis (MDD)	16; 76.19%	18; 85.71%	34; 80.92%	0.69 ^b^	0.095
Age (years; mean ± SD)	68.71 (5.32)	68.57 (5.54)	68.64 (5.36)	0.93 ^a^	−0.026
Age at onset (years; mean ± SD)	45.33 (16.73)	44.38 (13.44)	44.86 (14.99)	0.92 ^a^	−0.063
Current episode duration (weeks; mean ± SD)	7.29 (5.71)	7.05 (4.85)	7.17 (5.23)	0.92 ^a^	−0.045
Antidepressants (SSRIs; SNRIs; TCAs)	11; 3; 7 52.38%; 14.29%; 33.33%	14; 3; 4 66.67%; 14.29%; 19.05%	25; 6; 11 59.52%; 14.29%; 26.19%	0.55 ^b^	0.145
Mood stabilizers (yes)	3; 14.29%	4; 19.05%	7; 16.67%	1.00 ^b^	−0.128
Antipsychotics (yes)	5; 23.81	4; 19.05%	9; 21.43%	1.00 ^b^	0.086
Benzodiazepines (yes)	16; 76.19%	17; 80.95%	33; 78.57%	0.73 ^b^	−0.030
HDRS21 at baseline	23.95 (4.08)	23.24 (5.44)	23.60 (4.76)	0.69 ^a^	−0.137
HDRS21 at 2 weeks	17.38 (6.84)	9.14 (4.99)	13.26 (7.23)	0.0001 ^a^	−1.376
HDRS21 at 1 month	13.71 (7.94)	5.81 (4.78)	9.76 (7.61)	0.001 ^a^	−1.206
GAF at baseline	55.86 (7.26)	54.86 (6.76)	55.36 (6.95)	0.88 ^a^	−0.143
GAF at 1 month	73.10 (7.52)	71.48 (9.86)	72.29 (8.70)	0.67 ^a^	−0.1846

^a^ Mann–Whitney; ^b^ Chi-Squared; SMD: standardized mean difference; rTMS: repetitive Transcranial Magnetic Stimulation; tDCS: transcranial Direct Current Stimulation; MDD: Major Depressive Disorder; SD: standard deviation; HDRS21: 21-item Hamilton Depression Rating Scale; GAF: Global Assessment of Functioning; SSRIs: Selective Serotonin Reuptake Inhibitors; SNRIs: Serotonin-Norepinephrine Reuptake Inhibitors; TCAs: Tricyclic Antidepressants.

## Data Availability

The data presented in this study are available on request from the corresponding author. The data are not publicly available due to privacy and ethical restrictions.

## Abbreviations

The following abbreviations are used in this manuscript:

NIBS	Non-invasive brain stimulation
rTMS	repetitive Transcranial Magnetic Stimulation
tDCS	transcranial Direct Current Stimulation
HDRS21	21-item Hamilton Depression Rating Scale
GAF	Global Assessment of Functioning
LLD	Late-life depression
MDD	Major Depressive Disorder
MDEs	Major Depressive Episodes
BD	Bipolar Disorder
DSM-5	Diagnostic and Statistical Manual of Mental Disorders, 5th edition
DLPFC	Left dorsolateral prefrontal cortex
SSRIs	Selective Serotonin Reuptake Inhibitors
SNRIs	Serotonin-Norepinephrine Reuptake Inhibitors
TCAs	Tricyclic Antidepressants
SMDs	Standardized mean differences
