# Correction: Prato et al. Non-Invasive Brain Stimulation in Older Inpatients with Depression: A Real-World Comparison of Repetitive Transcranial Magnetic Stimulation (rTMS) and Transcranial Direct Current Stimulation (tDCS) on Depressive Symptoms and Functional Recovery. *Biomedicines* 2026, *14*, 650

**DOI:** 10.3390/biomedicines14091890

**Published:** 2026-08-25

**Authors:** Michele Prato, Barbara Barbini, Filippo Frizzi, Matteo Carminati, Greta Verri, Sebastiano Busseni Cantoni, Thomas Kafka, Raffaella Zanardi, Cristina Colombo

**Affiliations:** 1 Department of Clinical Neurosciences, Vita-Salute San Raffaele University, 20127 Milan, Italy; prato.michele@hsr.it (M.P.); frizzi.filippo@hsr.it (F.F.); verri.greta@hsr.it (G.V.); busseni.sebastiano@hsr.it (S.B.C.); t.kafka@studenti.unisr.it (T.K.); zanardi.raffaella@hsr.it (R.Z.); colombo.cristina@hsr.it (C.C.); 2 Department of Psychiatry, Mood Disorder Unit, Scientific Institute IRCCS Ospedale San Raffaele, 20127 Milan, Italy; carminati.matteo@hsr.it

## Error in Tables

In the original publication [1], there were mistakes in Table 1. The variable “Current episode duration” is expressed in months; the header and the associated column total have been corrected accordingly. A column total and one group percentage have also been corrected, and the superscript letters indicating the statistical test applied to each variable have been reassigned to the correct rows. Minor typographical corrections were also made. These changes concern the presentation of the data only and do not affect the per-group values, the statistical analyses, the results reported in the main text, or the conclusions of the article. The corrected Table 1 appears below. 

In the original publication [1], there were mistakes in Table 2. The values reported for HDRS21 at 2 weeks and at 1 month were inadvertently transposed between the two treatment columns and now appear in the correct columns; the matched-sample composition, the group totals, and the standardized mean differences are unchanged. As in Table 1, “Current episode duration” is expressed in months, and minor typographical and rounding corrections were made. The corrected Table 2 appears below. 

The authors state that the scientific conclusions are unaffected. This correction was approved by the Academic Editor. The original publication has also been updated.

## Figures and Tables

**Table 1:** Summary of sociodemographic and clinical variables of the sample.

	rTMS (*n* = 48)	tDCS (*n* = 56)	Total (*n* = 104)	*p*-Value	SMD
Sex (male)	12; 25.00%	7; 12.50%	19; 18.27%	0.16 ^b^	0.434
Diagnosis (MDD)	38; 79.17%	47; 83.93%	85; 81.73%	0.71 ^b^	−0.002
Age (years; mean ± SD)	65.29 ± 5.21	72.39 ± 5.75	69.12 ± 6.53	<0.001 ^a^	−1.311
Age at onset (years; mean ± SD)	43.30 ± 14.66	47.48 ± 15.19	45.93 ± 15.05	0.21 ^a^	−0.205
Current episode duration (months; mean ± SD)	6.64 ± 4.22	5.31 ± 4.31	5.92 ± 4.30	0.05 ^a^	0.379
Antidepressants (SSRIs; SNRIs; TCAs)	28; 9; 11 58.33%; 18.75%; 22.92%	29; 14; 13 51.79%; 25.00%; 23.21%	57; 23; 24 54.81%; 22.12%; 23.08%	0.34 ^b^	0.308
Mood stabilizers (yes)	12; 25.00%	8; 14.29%	20; 19.23%	0.26 ^b^	0.102
Antipsychotics (yes)	12; 25.00%	6; 10.70%	18; 17.31%	0.10 ^b^	0.212
Benzodiazepines (yes)	37; 77.08%	45; 80.36%	82; 78.85%	0.69 ^b^	−0.020
HDRS21 at baseline	23.47 ± 4.23	24.84 ± 4.59	24.34 ± 4.49	0.22 ^a^	−0.495
HDRS21 at 2 weeks	9.00 ± 4.39	18.11 ± 6.87	14.79 ± 7.49	<0.001 ^a^	−1.449
HDRS21 at 1 month	4.84 ± 3.87	13.32 ± 8.10	10.24 ± 7.98	<0.001 ^a^	−1.069
GAF at baseline	55.13 ± 6.42	56.27 ± 6.64	55.74 ± 6.53	0.56 ^a^	−0.163
GAF at 1 month	72.58 ± 8.49	73.36 ± 7.09	73.00 ± 7.74	0.74 ^a^	−0.020

^a^ Mann–Whitney; ^b^ Chi-Squared; SMD: standardized mean difference; rTMS: repetitive Transcranial Magnetic Stimulation; tDCS: transcranial Direct Current Stimulation; MDD: Major Depressive Disorder; SD: standard deviation; HDRS21: 21-item Hamilton Depression Rating Scale; GAF: Global Assessment of Functioning; SSRIs: Selective Serotonin Reuptake Inhibitors; SNRIs: Serotonin-Norepinephrine Reuptake Inhibitors; TCAs: Tricyclic Antidepressants.

**Table 2:** Summary of sociodemographic and clinical variables of the sample after propensity-based matching.

	rTMS (*n* = 21)	tDCS (*n* = 21)	Total (*n* = 42)	*p*-Value	SMD
Sex (male)	4; 19.05%	3; 14.29%	7; 16.67%	1.00 ^b^	0.128
Diagnosis (MDD)	16; 76.19%	18; 85.71%	34; 80.95%	0.69 ^b^	0.095
Age (years; mean ± SD)	68.71 (5.32)	68.57 (5.54)	68.64 (5.36)	0.93 ^a^	−0.026
Age at onset (years; mean ± SD)	45.33 (16.73)	44.38 (13.44)	44.86 (14.99)	0.92 ^a^	−0.063
Current episode duration (months; mean ± SD)	7.29 (5.71)	7.05 (4.85)	7.17 (5.23)	0.92 ^a^	−0.045
Antidepressants (SSRIs; SNRIs; TCAs)	11; 3; 7 52.38%; 14.29%; 33.33%	14; 3; 4 66.67%; 14.29%; 19.05%	25; 6; 11 59.52%; 14.29%; 26.19%	0.55 ^b^	0.145
Mood stabilizers (yes)	3; 14.29%	4; 19.05%	7; 16.67%	1.00 ^b^	−0.128
Antipsychotics (yes)	5; 23.81%	4; 19.05%	9; 21.43%	1.00 ^b^	0.086
Benzodiazepines (yes)	16; 76.19%	17; 80.95%	33; 78.57%	0.73 ^b^	−0.030
HDRS21 at baseline	23.95 (4.08)	23.24 (5.44)	23.60 (4.76)	0.69 ^a^	−0.137
HDRS21 at 2 weeks	9.14 (4.99)	17.38 (6.84)	13.26 (7.23)	0.0001 ^a^	−1.376
HDRS21 at 1 month	5.81 (4.78)	13.71 (7.94)	9.76 (7.61)	0.001 ^a^	−1.206
GAF at baseline	55.86 (7.26)	54.86 (6.76)	55.36 (6.95)	0.88 ^a^	−0.143
GAF at 1 month	73.10 (7.52)	71.48 (9.86)	72.29 (8.70)	0.67 ^a^	−0.185

^a^ Mann–Whitney; ^b^ Chi-Squared; SMD: standardized mean difference; rTMS: repetitive Transcranial Magnetic Stimulation; tDCS: transcranial Direct Current Stimulation; MDD: Major Depressive Disorder; SD: standard deviation; HDRS21: 21-item Hamilton Depression Rating Scale; GAF: Global Assessment of Functioning; SSRIs: Selective Serotonin Reuptake Inhibitors; SNRIs: Serotonin-Norepinephrine Reuptake Inhibitors; TCAs: Tricyclic Antidepressants.
